# Prediction of the service life of surgical instruments from the surgical instrument management system log using radio frequency identification

**DOI:** 10.1186/s12913-019-4540-0

**Published:** 2019-10-15

**Authors:** Takeki Yoshikawa, Eizen Kimura, Emi Akama, Hiromi Nakao, Toshihiro Yorozuya, Ken Ishihara

**Affiliations:** 1 Surgical division, Ehime University Hospital, Shitsukawa, Toon city, Ehime 791-0295 Japan; 2 Department of Medical Informatics, Ehime University Graduate School of Medicine, Shitsukawa, Toon city, Ehime 791-0295 Japan; 3 Japanese Red Cross Wakayama Medical Center, 4-20 Komatsubara-dori, Wakayama City, Wakayama, 640-8558 Japan; 4 Department of Anesthesia and Perioperative Medicine, Ehime University Graduate School of Medicine, Shitsukawa, Toon city, Ehime 791-0295 Japan; 5 Department of Medical Informatics, Ehime University Graduate School of Medicine, Shitsukawa, Toon city, Ehime 791-0295 Japan

**Keywords:** RFID, Tracking data, Probability model, Service life of surgical instruments, Prevention of medical accidents

## Abstract

**Background:**

Bar code- or radio frequency identification (RFID)-based medical instrument management systems have gradually been introduced in the field of surgical medicine for the individual management and identification of instruments. We hypothesized that individual management of instruments using RFID tags can provide previously unavailable information, particularly the precise service life of an instrument. Such information can be used to prevent medical accidents caused by surgical instrument failure. This study aimed to predict the precise service life of instruments by analyzing the data available in instrument management systems.

**Methods:**

We evaluated the repair history of instruments and the usage count until failure and then analyzed the data by the following three methods: the distribution of the instrument usage count was determined, an instrument failure probability model was generated through logistic regression analysis, and survival analysis was performed to predict instrument failure.

**Results:**

The usage count followed a normal distribution. Analysis showed that instruments were not used uniformly during surgery. In addition, the Kaplan–Meier curves plotted for five types of instruments showed significant differences in the cumulative survival rate of different instruments.

**Conclusions:**

The usage history of instruments obtained with RFID tags or bar codes can be used to predict the probability of instrument failure. This prediction is significant for determining the service life of an instrument. Implementation of the developed model in instrument management systems can help prevent accidents due to instrument failure. Knowledge of the instrument service life will also help in developing a purchase plan for instruments to minimize wastage.

## Background

Major medical accidents are caused by human error [1], which can be mitigated with double-checking by more than one person or the use of computerized order entry. For example, medication errors caused by the incorrect combination of drugs and patients are mostly due to human error [2–4]. Bar codes [5, 6] or radiofrequency identification (RFID) tags [7, 8] have been used to identify patients and drugs in order to avoid medication errors. Studies have also investigated the prevention of medical accidents through using verification systems for drug identification, blood transfusion products, and medical instruments as individual healthcare materials [9–11].

Analysis of log data such as the location and use history of individual medical resources, e.g., drugs, which enable follow-up, is considered valuable for the retrospective determination of the cause of medical accidents [12]. However, conducting follow-up surveys for instruments using the log data has been difficult because of the limitation of individual management of surgical instruments.

As RFID tag information can be read wirelessly, medical staff handling surgical instruments can easily read information obtained from RFID tags attached to surgical instruments for individual management [13]. However, washing and sterilization of the instruments at high temperatures can damage or contaminate the integrated circuit (IC) chip in the RFID tag and thereby limit the readability of RFID tag information. Therefore, RFID tags have not been adopted extensively in the management of steel surgical instruments. Ceramic-coated RFID tags attached to or embedded in surgical instruments can resolve this problem [14, 15]. Although the RFID-based medical instrument management system has gradually been introduced in the field of surgical medicine [16], the system has yet to gain popularity. In 2011, we developed an RFID-based medical instrument management system using ceramic-coated RFIDs [17].

The instrument management system facilitates individual management and identification of instruments by bar codes or RFID tags as well as collection of information about instrument use history and location (i.e., tracking data). We hypothesized that the individual management of instruments using RFID tags or bar codes can provide new insights into surgical instrument usage. For example, we anticipate that the precise service life of instruments can be determined with these data, which can be then implemented to prevent medical accidents caused by surgical instrument failure. Prior to this study, the precise service life of instruments could not be determined because of the lack of clear data on the frequency of usage and fault occurrence. Therefore, this study aimed to predict the precise service life of instruments based on evidence obtained by analyzing the data accumulated in the instrument management system we developed. Today, the use of RFID tags and bar codes can enable the identification of individual surgical instruments. Therefore, the aim of this study was to evaluate the potential of using RFID tags to gain useful information based on the identification of individual surgical instruments. To the best of our knowledge, a similar analysis that is concerned with surgical instruments actually used in clinical practice, has not been published yet.

## Methods

### Target data

We developed a surgical instrument traceability system using RFID tags [17] and deployed the system at the Japanese Red Cross Wakayama Medical Center in July 2013. We retrieved the instrument-related data accumulated in the system from September 1, 2013 to April 30, 2017. The data from all surgeries that were performed in 15 surgical departments in the center (general surgery, ophthalmology, otolaryngology, orthopedic surgery, gynecology, cardiovascular surgery, pediatric cardiac surgery, urology, neurosurgery, thoracic surgery, plastic surgery, pediatric surgery, breast surgery, emergency department, and dental and oral surgery) were included. There have been 34,390 surgical operations during data collection. No medical accidents related to surgical instruments occurred at the medical center during this period. System errors occurred on an average of 1.6 cases per month. We removed the patients’ personal information from the acquired data through unlinkable anonymization. We have not used any patient data in this study. This study has been approved by the Ethics Committee of Ehime University Hospital.

### RFID-based surgical instrument traceability system

#### System structure

**Fig. 1:**
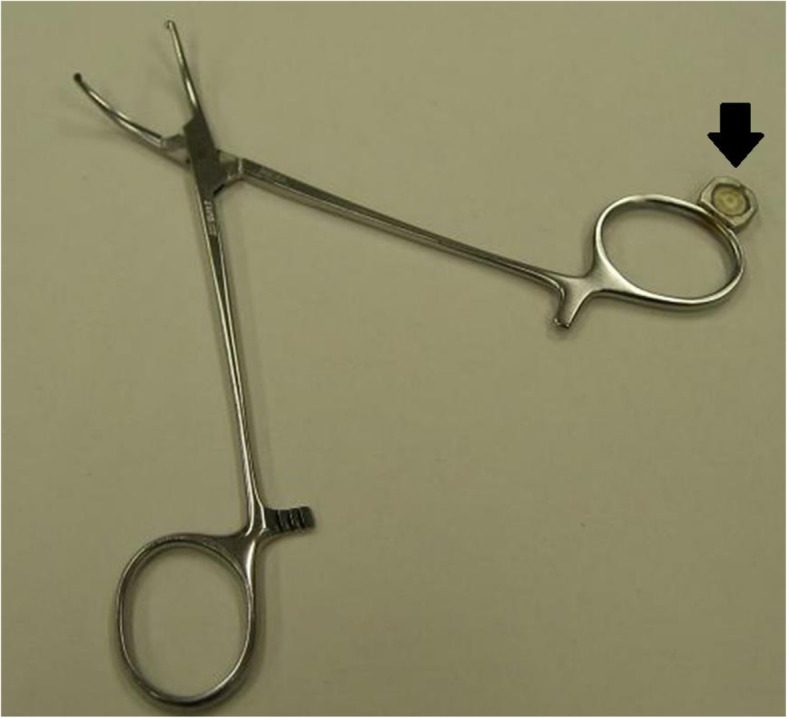
Instrument with a ceramic-coated RFID tag. The arrow shows the ceramic-coated RFID tag welded to the instrument

The traceability system that we developed comprises three elements. The first is a ceramic-coated RFID tag [18], which is attached to the instrument by welding (indicated by the arrow in Fig. 1). Every RFID tag has a unique ID that is adopted as the instrument’s ID. The second element is an RFID reader that wirelessly reads the ID in the RFID tag. The reader can read the information using 13.56 MHz radio frequency wave and within the upper 20 cm of the instrument. The third element is a surgical instrument management program installed on a personal computer connected to the RFID reader. We assign IDs to each computer and collect information on the location. Using the computer program, the staff members routinely record the history of instrument preparation before surgery, the number of instruments used during surgery, and the number and type of instruments used after surgery. When an instrument ID is read from the RFID tag, the program simultaneously records the time, the name of the staff member logged into the computer, and the location where the RFID tag is read. This information is stored for use in the information model developed to collect the information needed for a retrospective study, as described in the next section [19].

#### Entity-relationship diagram of the database for the traceability system

**Fig. 2:**
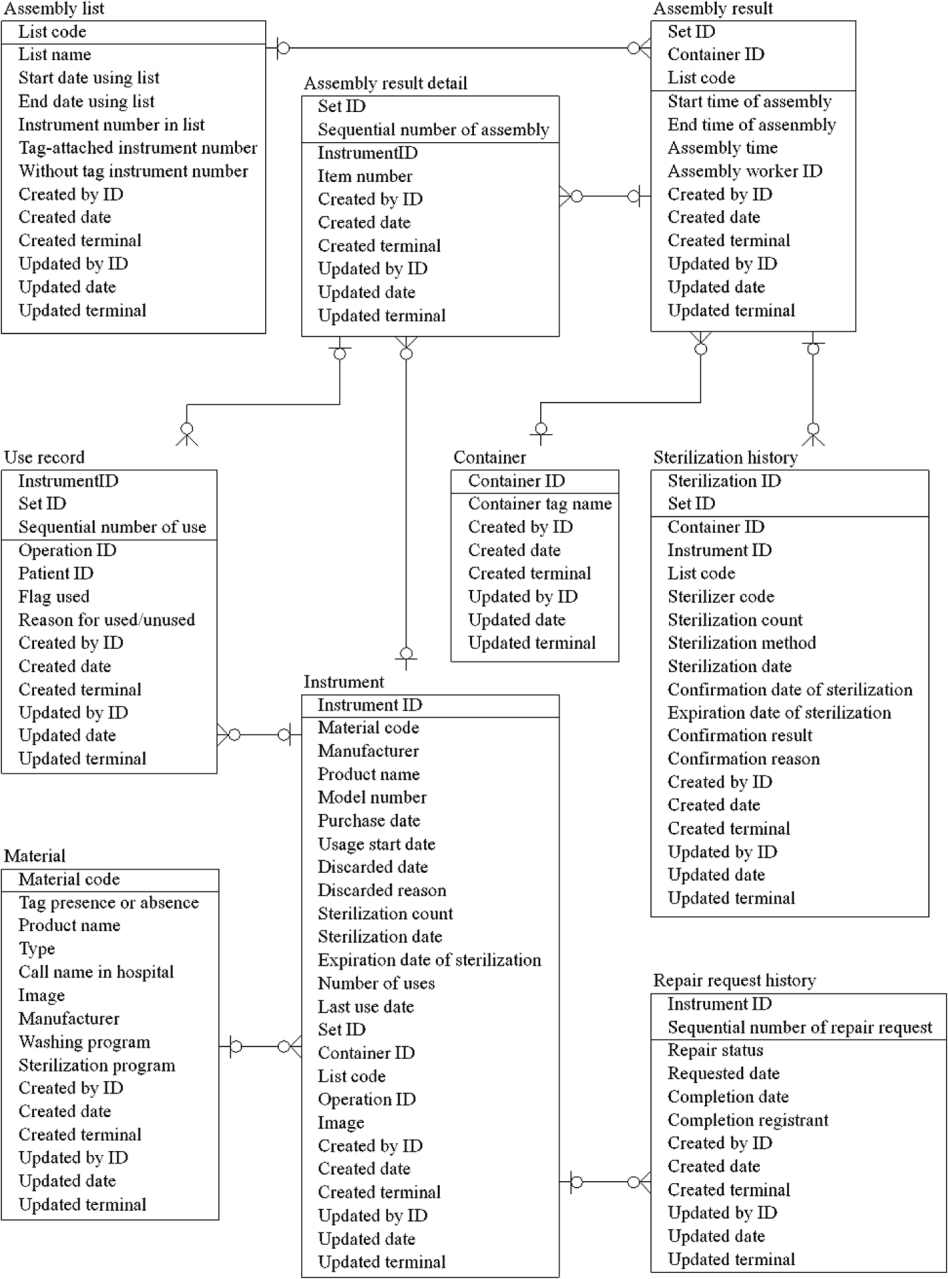
Entity-relationship diagram of the database for this system

We designed the database for this system based on an information model. Here, we present part of the entity-relationship diagram (ERD) of this system, shown in Fig. 2. An ERD is used to describe the structure of a database [20]. In this analysis, we extracted the “usage count” from “Use record” and “Repair request record” by “InstrumentID.” “Use record” contains information about when and in which operation each instrument is used. We also collected information about the instrument ID, set ID, and type of operation performed. Because surgeons do not always use all prepared instruments in the operative field during surgery, we tracked the use of each instrument during surgery. For example, in one case, only 5 of 10 pairs of sterilized forceps were actually used during surgery. The “repair request record” includes data on failed instruments, persons requesting repairs, repair request dates, repair request details, and repair completion dates.

#### Assembly

Assembly is a preoperative task during which the staff prepares the instruments that will be used in surgery. The staff members assemble washed instruments according to a preliminary list showing the numbers and types of instruments needed for surgery. The collected instruments are then placed in a container for sterilization.

In our system, assembly is performed using an electronic list displayed on the system. Referring to the list, the staff member passes the instruments over the RFID reader, confirms the result, then matches the RFID tag with the list, and places the instrument into the container.

An RFID tag is attached to the container, and the ID of the RFID tag is regarded as the container ID. The “set” comprises the container and the instruments placed in the container. A set ID is allocated to each set and linked with the container ID and the IDs of the instruments in the set. The set ID is accompanied by assembly-related information (i.e., assembly time, location, and staff). This assembly-related information is recorded in the database.

### Data analysis

We analyzed the repair history of instruments. We used the parameter “usage count,” which is the number of times an instrument is used until fault occurrence. We performed three types of analysis. We assessed the distribution of the usage count for the instruments (analysis I), created an instrument failure probability model (analysis II), and performed survival analysis to assess instrument failure (analysis III).

Statistical analyses were performed using SPSS software (ver. 22). The significance level was set at *p* < 0.05.

#### Analysis I: distribution of the usage count of instruments (distribution analysis)

To visualize the distribution of the usage counts and usage rate for instruments of the same type, we used the history data for Cooper scissors, which contain repair records. Usage rate is calculated as (usage count)/(sterilization count); this is because some sterilized instruments may not be used during a surgical procedure, as mentioned above.

We assumed that there was a bias in the usage count for instruments because the “Assembly process” showed no instrument replacements; that is, only specific instruments were used. Therefore, we tested the normality of the two distributions using the Kolmogorov–Smirnov test.

#### Analysis II: logistic regression analysis (instrument failure probability model)

We conducted a follow-up study with the data of instrument use, repair request, and instrument discard history. With the aim of estimating the durability period for each instrument, we calculated the incidence of instrument failure for each instrument type. For this analysis, we selected the Cooper scissors, which had the highest number of repair requests, and created an instrument failure probability model.

#### Extraction of instrument repair history data

We collected information about the instrument repair history data, including data on failed instruments, repair request details (i.e., the reason for repair), and usage count (before the failed instrument was repaired).

#### Instrument failure probability model

We created a failure probability model from the history data of 136 Cooper scissors for which repairs had been requested. We considered the issuance of a repair request as the occurrence of instrument failure. Because the occurrence of failure is a dependent variable expressed with a binary number, we performed logistic regression analysis with the parameter usage count as an independent variable [21]. We defined the regression curve as $y=\frac{1}{1+{e}^{-\left({b}_0+\sum {b}_i{x}_i\right)}}$ [21], where x is the covariate, y is the probability, b is the partial regression coefficient, and b_0_ is a constant. Finally, we evaluated the model by calculating the coefficient of determination and the *p*-value for the Hosmer–Lemeshow test.

#### Analysis III: Kaplan–Meier survival analysis for instrument failure

We performed Kaplan–Meier survival analysis on data for two types of surgical scissors (Cooper; *N* = 35, Metzenbaum; *N* = 28) and three types of forceps (Kelly; *N* = 20, Kocher; *N* = 58, Pean; *N* = 28) using the log-rank test. We plotted a Kaplan–Meier curve using the repair history data and then calculated the cumulative survival rates of every instrument from the usage count when the instruments failed.

## Results

### Analysis I: distribution of the usage count of instruments (distribution analysis)

**Fig. 3:**
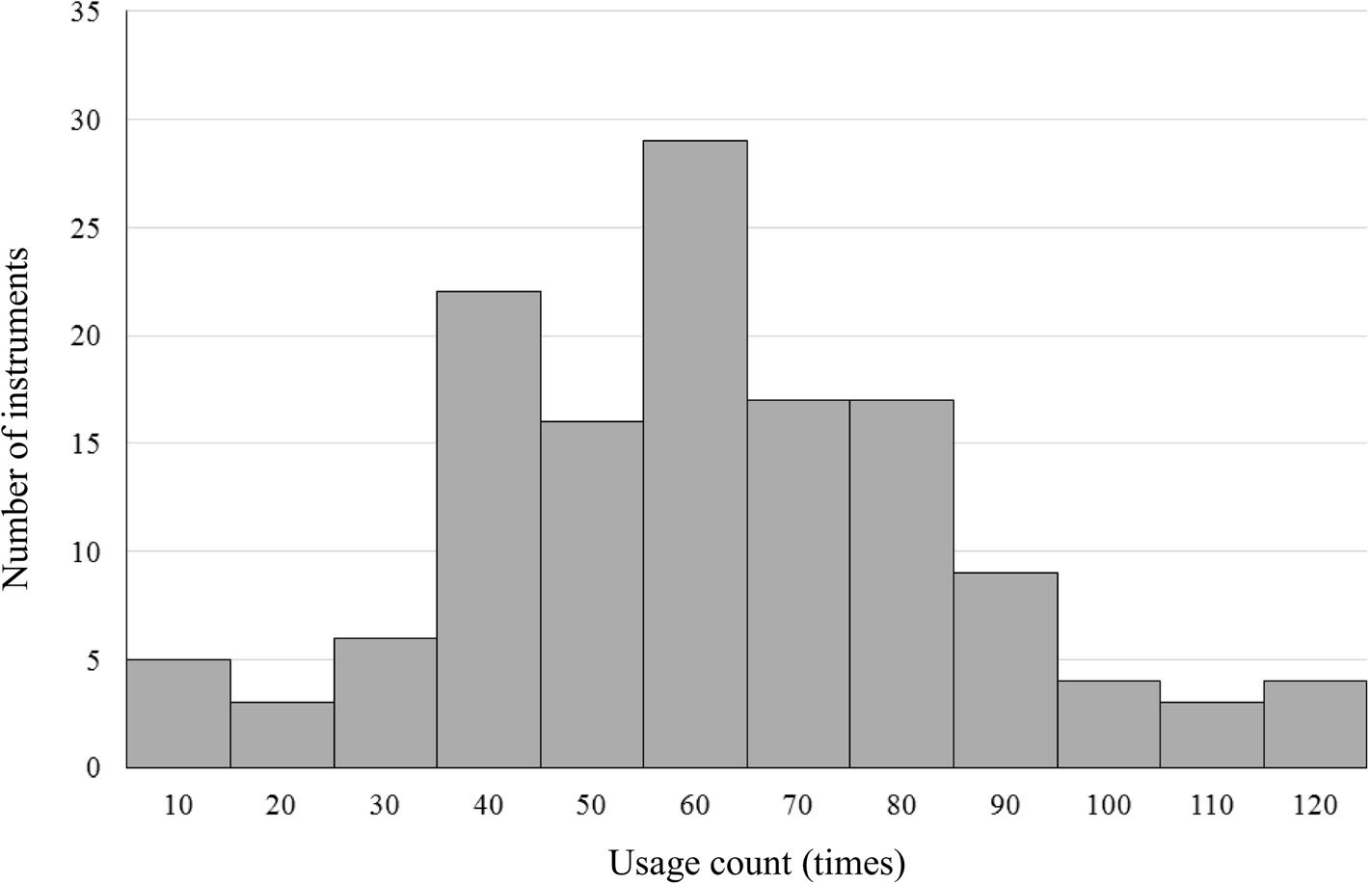
Distribution of the usage count. The histogram of the usage count for the 136 Coopers is plotted, with the x-axis showing the usage count with bins representing ten uses and the y-axis showing the number of instruments at each value of x

**Fig. 4:**
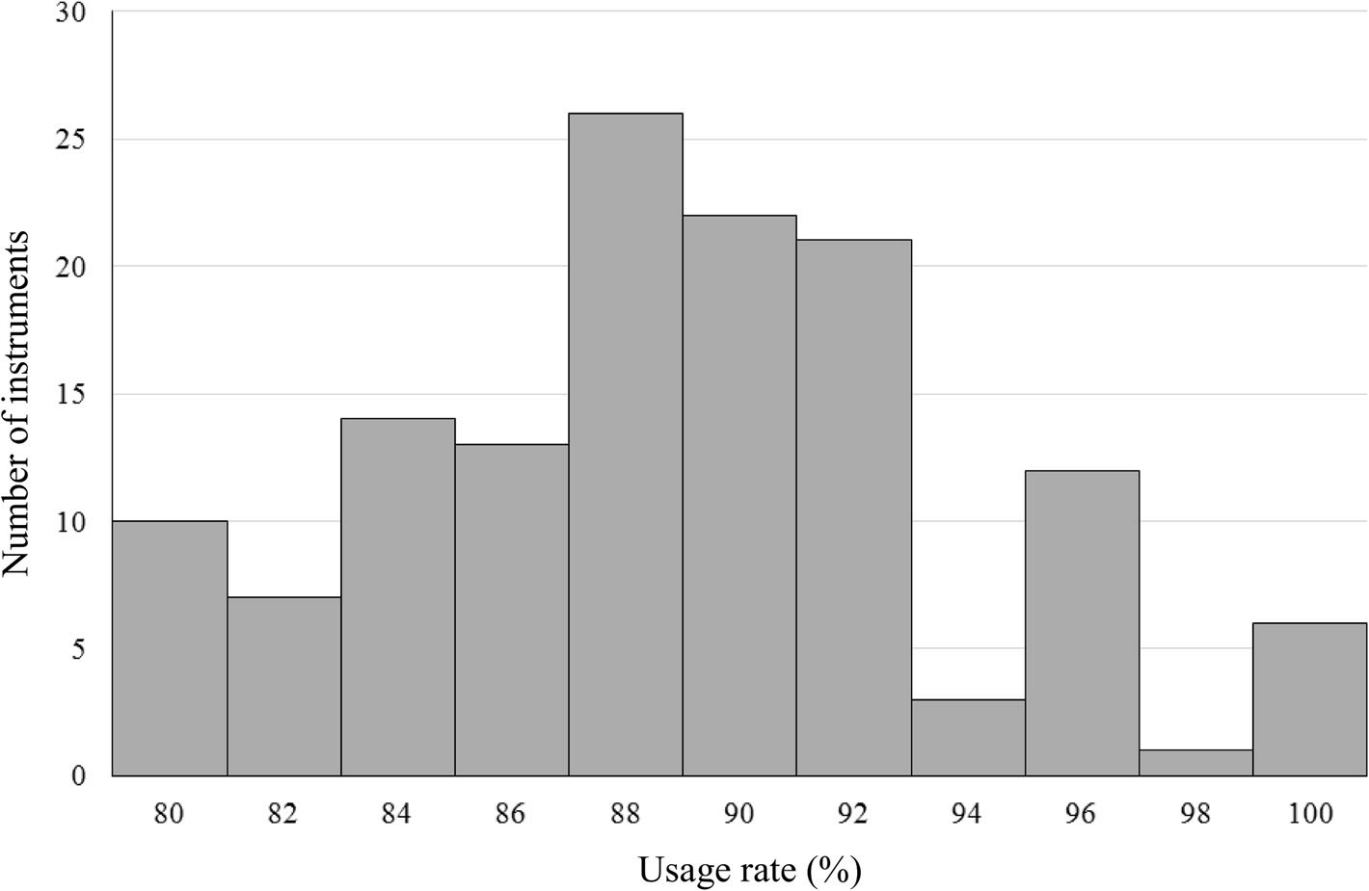
Distribution of the usage rate. The histogram of the usage rate for the 136 Coopers is plotted, with the x-axis showing the usage count with bins representing 2% and the y-axis showing the number of instruments at each value of x

Figure 3 shows the histogram of the usage count for the 136 Coopers. The *x*-axis shows the usage count with bins representing 10 uses, while the *y*-axis shows the number of instruments at each value of *x*. The Kolmogorov–Smirnov test indicates that the usage count follows a normal distribution (*p* = 0.2). Therefore, surgical instruments were not used uniformly throughout the study period. Figure 4 shows the histogram of the usage rate for the 136 Coopers. The *x*-axis shows the usage count with bins representing 2%, while the *y*-axis shows the number of instruments at each value of *x*. The Kolmogorov–Smirnov test indicates that the usage count follows a normal distribution (*p* = 0.098). Therefore, the ratio of the usage and sterilization counts is not uniform, indicating that not all sterilized instruments were used during surgery.

### Analysis II: logistic regression analysis (instrument failure probability model)

**Fig. 5:**
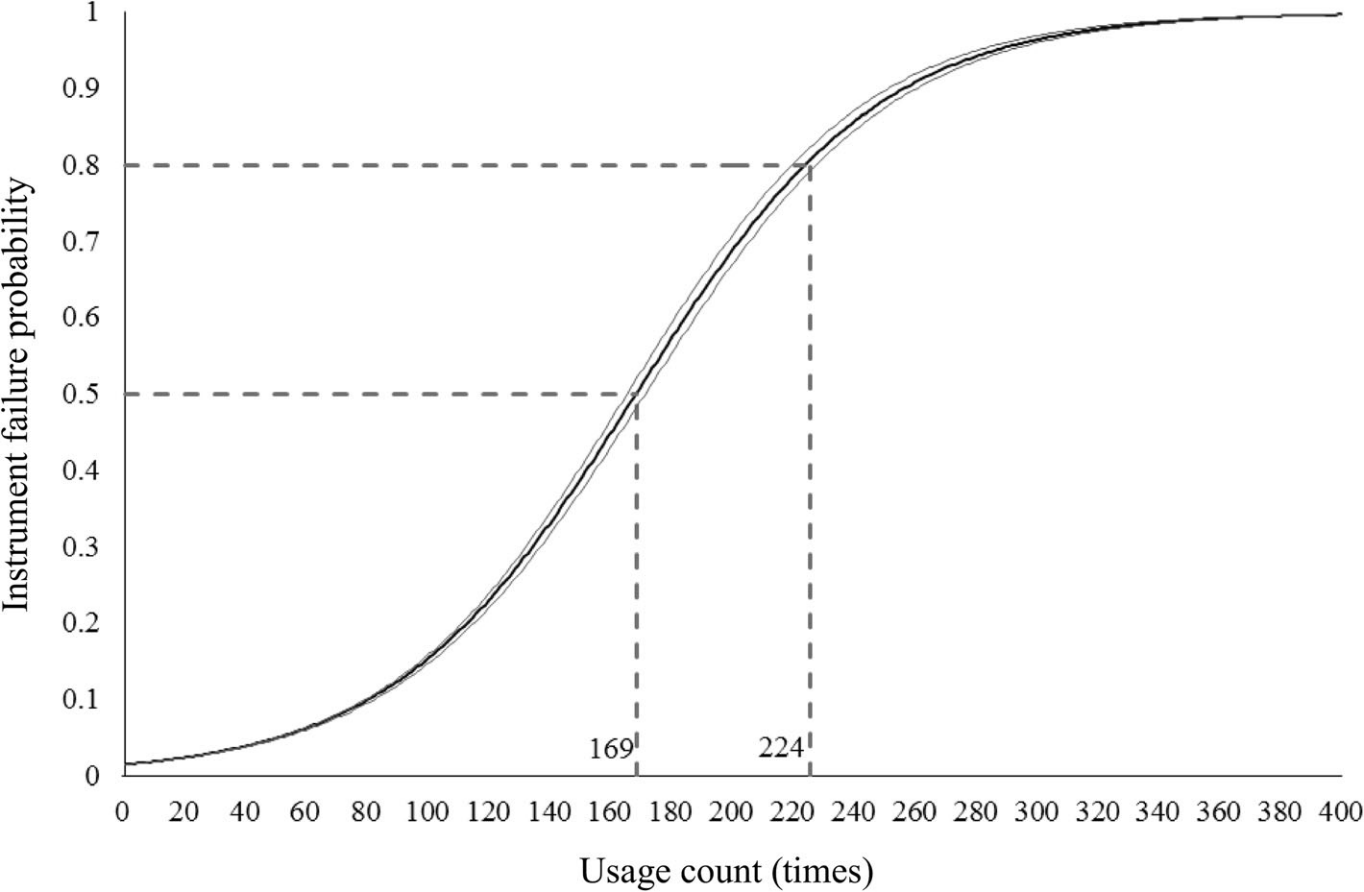
Regression curve for failure probability of Cooper scissors. The regression curve and its 95% confidence interval are plotted for the probability of failure (*y*-axis) against the usage count (*x*-axis)

Table 1 shows the results of logistic regression analysis of the failure probability for the 136 Coopers. The instruments were used multiple times per surgery. An increase in the usage count caused a 1.026-fold increase in the risk of instrument failure occurrence. The coefficient of determination is 0.032 and the *p*-value for the Hosmer–Lemeshow test is 0.117. Figure 5 shows the regression curve and its 95% confidence interval. The probability of failure (*y*-axis) is plotted against the usage count (*x*-axis). When the usage count exceeded 169, the probability of failure exceeded 0.5. Furthermore, when the usage count exceeded 224, the probability of failure exceeded 0.8.

**Table 1:** Results of logistic regression analysis

	B	SE	p	Odds ratio
Usage count	0.025	0.004	< 0.001	1.026
Constant	−4.213	0.163	< 0.001	0.015

Results of logistic regression analysis for the failure probability of Cooper scissors

### Analysis III: Kaplan–Meier survival analysis of instrument failure

**Fig. 6:**
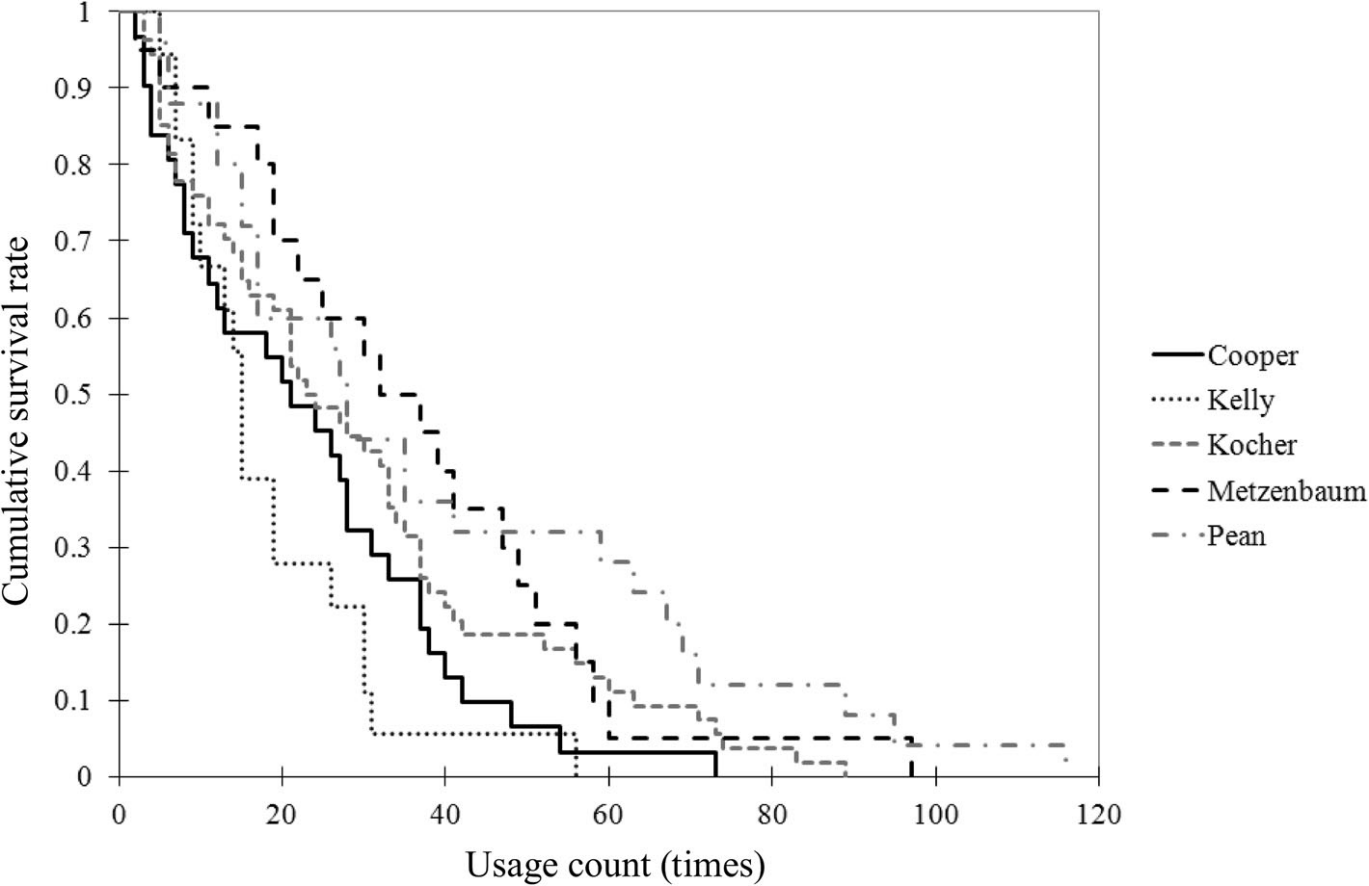
Kaplan–Meier curves for five types of instruments**.** The Kaplan–Meier curves for five types of instruments are plotted for the cumulative survival rate (y-axis) against the usage count (x-axis)

Figure 6 shows the Kaplan–Meier curves for five types of instruments. The cumulative survival rate (*y*-axis) is plotted against the usage count (*x*-axis). Table 2 shows the results of the log-rank test and the *p*-value pair for each instrument. There were significant differences between (1) Kelly and Kocher, Metzenbaum, and Pean, (2) Cooper and Metzenbaum, and (3) Cooper and Pean.

**Table 2:** Log-rank test (*p*-value)

	Cooper	Kelly	Kocher	Metzenbaum	Pean
Cooper		0.316	0.159	0.036	0.024
Kelly	0.316		0.017	0.003	0.004
Kocher	0.159	0.017		0.328	0.106
Metzenbaum	0.036	0.003	0.328		0.559
Pean	0.024	0.004	0.106	0.559	

Results of the log-rank test and the *p*-value pair for each instrument

## Discussion

### Non-uniformity in the distribution of the usage count for the instruments

We confirmed that there was non-uniformity in the usage count among individual Cooper scissors. After the first surgical operation, the same instruments tend to be used repeatedly, and the combination of instruments is unlikely to be rearranged. If all Coopers are used the same number of times, we can estimate the service life of the instrument by simply dividing the total period between the start of use and disposal with the total usage counts for all instruments. In reality, however, the usage counts for individual instruments are not uniform; therefore, individual instruments should be tracked for accurate prediction of the instrument’s service life.

This kind of non-uniformity can lead to economic loss (as some instruments are left unused). In addition, incorrect prediction of an instrument’s service life may fail to prevent an accident due to a broken piece of the failed instrument during surgery. Previously, the identification and follow-up of individual surgical instruments and the replacement of instruments based on the history of use were not possible. We can identify individual instruments and obtain the usage count of each individual instrument using bar codes or RFID tags. Furthermore, by implementing an instrument failure probability model in this system in the future, we can be develop an effective warning system that can indicate either replacement or disposal of instruments before the occurrence of failure, and thereby prevent accidents due to broken instruments during surgery.

### Instrument failure probability model

We created the model that estimates the probability of instrument failure based on the usage count (results of analysis II). The model can be rendered more accurate with the inclusion of other factors. For example, although the manufacturers of surgical instruments perform endurance tests on the instruments, obtaining data about which instruments are used in practice is beneficial because of the inclusion of information such as the differences in the manner the instruments are used by surgeons, across departments, and across facilities. In this study, we used only the usage count for our analysis. However, data collected over a longer period with more categories can enhance the accuracy of the model. We believe this model will facilitate precise prediction of the service life of instruments.

### Frequency of differences in instrument failure among different types of instruments

Surgeons use Cooper scissors more frequently than Metzenbaum scissors to cut harder tissues. Moreover, surgeons use Cooper scissors, but not Metzenbaum scissors, to cut sutures and surgical drapes. Consequently, there was a significant usage count difference between Cooper and Metzenbaum scissors (*p* = 0.036). In contrast, the usage count difference between Kocher and Pean forceps (*p* = 0.106) was not significant. However, there were significant differences between Kelly and Kocher forceps (*p* = 0.017) and between Kelly and Pean forceps (*p* = 0.004). Surgeons use Kelly forceps for tissue ablation. Since Kelly forceps are dainty and elaborate, they tend to fail more frequently. We will obtain more information from surveying further types of instruments in the future.

### Future possibilities for individual management with RFID tags

We can obtain information about the use history of instruments, which was unavailable prior to the implementation of RFID tags and bar codes. Using this information, we can predict the probability of instrument failure, which is significant information for determining the service life of instruments and developing an instrument purchase plan that can reduce wastage.

We were able to acquire new information concerned with the surgical instruments using the tracking technology (RFID, bar code). This approach of using the new information may lead to more effective management of instruments in terms of prevention of medical errors, reduction of waste, and instrument purchase planning. As surgical operations need aseptic operations, we proposed that RFID tags are more suitable for surgical instruments than bar codes. However, identification of individual instruments is important rather than comparison of identification abilities between RFID tags and bar codes. In other words, once the instruments are identified, either RFID tags or bar codes can be acceptable as tracking tools. In the present study, we evaluated the potential of obtaining new information based on the tracking data, although we did compare the identification abilities between RFID tags and bar codes. Instruments equipped with RFID tags are significantly more expensive (~ 5 US dollars per instrument) than instruments without tags; however, more accurate cost management would be facilitated owing to the use of tags, and thus unnecessary costs concerned with instruments may be reduced. However, we believe that it is difficult to present merits in terms of costs of using tags compared with costs of introducing the system. Costs of medical litigation vary with cases, thereby making it difficult to assess effectiveness in preventing medical accidents in terms of cost. In this regard, we believe that the use of tags for surgical instruments has more merit than the use of surgical instruments without tags.

## Conclusions

We hypothesized that individual management of instruments using RFID tags can provide useful information. To correct non-uniform usage of instruments, individual devices should be tracked. Implementation of instrument management systems with the instrument failure probability model may help prevent accidents caused by instrument failure. The instrument failure probability model and survival analysis of instruments can help us determine the precise service life of instruments.

## Data Availability

The data that support the findings of this study are available from Ehime University but restrictions apply to the availability of these data, which were used under license for the current study, and so are not publicly available. Data are however available from corresponding author upon reasonable request and with permission of Ehime University.

## Abbreviations

APAMI: Asia Pacific Association for Medical Informatics
ERD: Entity-relationship diagram
IC: Integrated circuit
RFID: Radio Frequency Identification
